# Navigated bedside implantation of external ventricular drains with mobile health guidance: technical note and case series

**DOI:** 10.1007/s00701-024-05955-w

**Published:** 2024-02-10

**Authors:** Tarik Alp Sargut, Ulrich-Wilhelm Thomale, Matthias Schulz, Andreas Schaumann, Ulf Christoph Schneider, Simon Heinrich Bayerl, Philipp Spindler, Peter Vajkoczy, Kiarash Ferdowssian

**Affiliations:** 1 Department of Neurosurgery, Charité – Universitätsmedizin Berlin, Freie Universität Berlin and Humboldt-Universität zu Berlin, Berlin, Germany; 2 Division of Pediatric Neurosurgery, Charité – Universitätsmedizin Berlin, Freie Universität Berlin and Humboldt-Universität zu Berlin, Berlin, Germany; 3 Department of Neurosurgery, Cantonal Hospital of Lucerne, Lucerne, Switzerland

**Keywords:** Bedside EVD, Navigated EVD, ICP monitoring, CSF, Hydrocephalus, Mobile health

## Abstract

**Purpose:**

External ventricular drain (EVD) implantation is one of the fundamental procedures of emergency neurosurgery usually performed freehand at bedside or in the operating room using anatomical landmarks. However, this technique is frequently associated with malpositioning leading to complications or dysfunction. Here, we describe a novel navigated bedside EVD insertion technique, which is evaluated in a clinical case series with the aim of safety, accuracy, and efficiency in neurosurgical emergency settings.

**Methods:**

From 2021 to 2022, a mobile health–assisted navigation instrument (Thomale Guide, Christoph Miethke, Potsdam, Germany) was used alongside a battery-powered single-use drill (Phasor Health, Houston, USA) for bedside EVD placement in representative neurosurgical pathologies in emergency situations requiring ventricular cerebrospinal fluid (CSF) relief and intracranial pressure (ICP) monitoring.

**Results:**

In all 12 patients (8 female and 4 male), navigated bedside EVDs were placed around the foramen of Monro at the first ventriculostomy attempt. The most frequent indication was aneurysmal subarachnoid hemorrhage. Mean operating time was 25.8 ± 15.0 min. None of the EVDs had to be revised due to malpositioning or dysfunction. Two EVDs were converted into a ventriculoperitoneal shunt. Drainage volume was 41.3 ± 37.1 ml per day in mean. Mean length of stay of an EVD was 6.25 ± 2.8 days. Complications included one postoperative subdural hematoma and cerebrospinal fluid infection, respectively.

**Conclusion:**

Combining a mobile health–assisted navigation instrument with a battery-powered drill and an appropriate ventricular catheter may enable and enhance safety, accuracy, and efficiency in bedside EVD implantation in various pathologies of emergency neurosurgery without adding relevant efforts.

## Introduction

Insertion of an external ventricular drain (EVD) is one of the most frequently performed procedures in neurosurgery worldwide [22, 31]. EVD insertion is an essential part in emergency management of acute hydrocephalus, intracranial hypertension, cerebrospinal fluid (CSF) infections, and continuous intracranial pressure (ICP) monitoring or to instill medication. Traditionally, insertion of the EVD catheter is performed in the frontal, precoronal position with a manually twisted hand drill at bedside or in the operating room setting in freehand technique, guided by anatomical landmarks. These landmarks are mainly Kocher’s point, medial epicanthus or midpupillary line, and the external acoustic meatus [2–4]. Recently, multiple studies have reported relevant rates of malpositioned freehand EVDs between 3 and 60%, resulting in revision or reinsertion procedures in up to half of these cases [1, 20, 27]. Intracranial hemorrhage, CSF infections, and neurological deficits are additional complications that may be caused by EVD misplacement with either multiple puncture attempts or need of revision [13, 18]. To overcome the problems of incorrectly placed ventricular catheters, the usage of a variety of technical aids such as guiding protractors [10, 32], fluoroscopy or computer tomography (CT) [8, 9], frameless stereotaxy [14, 24], ultrasonography [11], neuroendoscopy [29], electromagnetic neuronavigation [17], robotics [16, 23], and smartphone-assisted guidance has been reported [6, 7, 19, 25]. To date, all these techniques are disadvantageous due to higher costs, longer procedure times, and more technical efforts including the necessity of taking the patient to the operating room, making bedside EVD implantation impossible. Furthermore, technical capabilities might not be equally available at all institutions. This may influence outcomes in neurocritical care, especially in patients with narrow ventricles and/or midline shift. We therefore report a novel technique of controlled and low effort bedside EVD implantation, with the use of a mobile health–assisted, ventricular catheter guide (Thomale Guide, Christoph Miethke, Potsdam, Germany) and an electric single-use power drill (Phasor Health, Houston, USA).

## Material and methods

In a prospective case series treated between 2021 and 2022, a ventricular catheter guide (Thomale Guide, Christoph Miethke, Potsdam, Germany) was used in combination with a battery-powered single-use drill (Phasor Health, Houston, USA), as opposed to the standard hand drill (CODMAN® Hand Drill, Integra LifeSciences, NJ, USA) with a compatible drill bit (Drill Kit, Raumedic, Helmbrechts, Germany) on site for bedside EVD placement, in adult and pediatric patients with representative neurosurgical pathologies in emergency situations at Campus Virchow-Klinikum and Benjamin Franklin of the Department of Neurosurgery at the Charité – Universitätsmedizin Berlin. This technique was consecutively applied in every patient by either one of two instructed resident physicians in postgraduate year 2 (junior) and year 4 (senior), respectively, when on call, without assistance by other medical staff.

### Preoperative planning

**Fig. 1:**
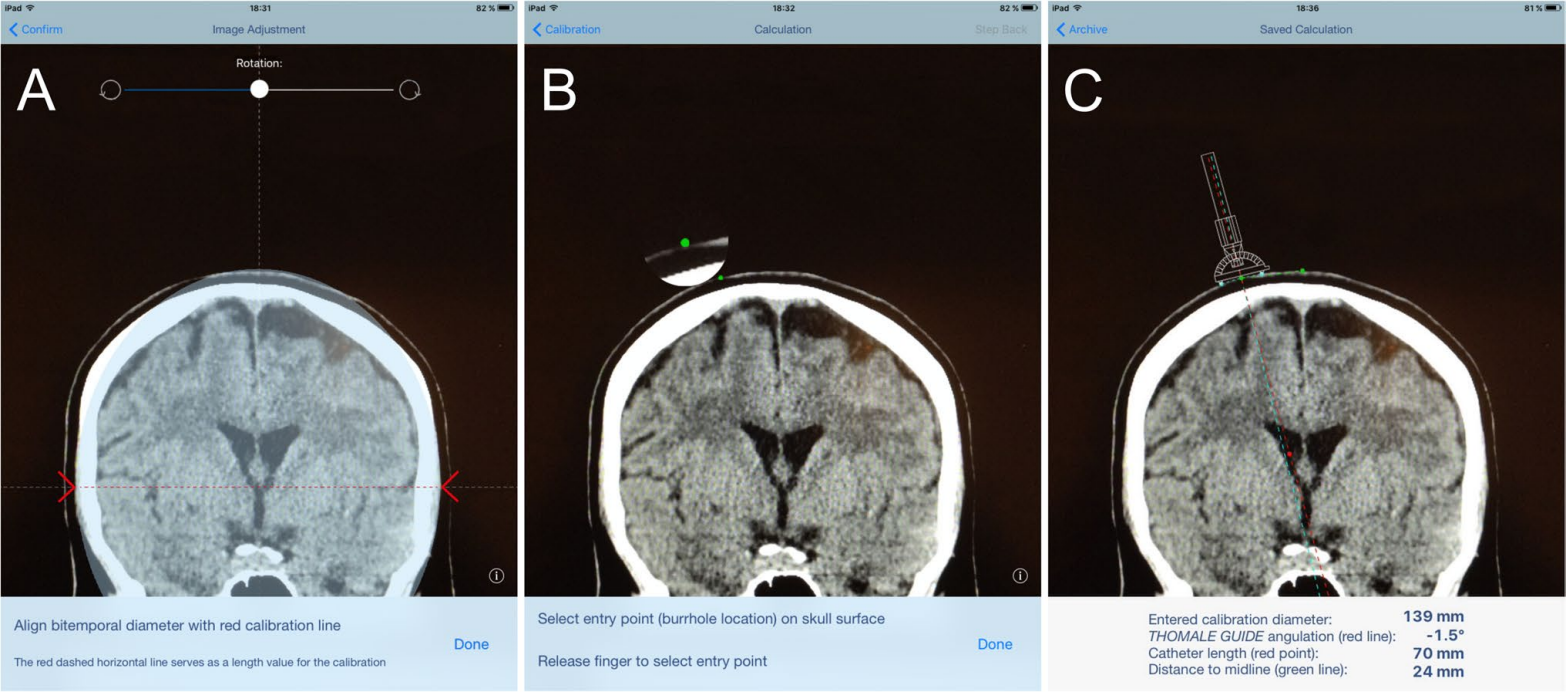
Illustrative example of trajectory planning. In-app view on an Apple iPad of a patient with narrow ventricles and traumatic brain injury (TBI). **A** Initially, an anterior coronal image is imported and adjusted in size to the image overlay. **B** The entry point and the guiding tool’s base are virtually placed on the scalp surface. **C** Then, the angulation is adjusted to place the tube’s direction towards the foramen of Monro in the ipsilateral ventricle (red dashed line), and the catheter length (dot on red dashed line) and distance to midline (dot on green dashed line) are measured, accordingly. Finally, all relevant calculations are presented

Application of the ventricular catheter guide (Thomale Guide, Christoph Miethke, Potsdam, Germany) was previously described for controlled implantation of ventricular shunt catheters [25, 26]. Most importantly, the catheter guide enables insertion of the ventricular catheter orthogonally to the sagittal plane with an individual, adjustable angle in coronal orientation in relation to calvarial slope [21]. The catheter trajectory is calculated in advance of EVD implantation by means of a mobile health app (iOS, iTunes: Thomale Guide App, Christoph Miethke, Potsdam, Germany) on a smartphone (iPhone, Apple, Cupertino, USA) or tablet computer (iPad, Apple, Cupertino, USA) (Fig. 1). As the ventricular catheter is placed bedside and percutaneously through a straight incision, in contrast to the standard application of the Thomale Guide placed on the skull via a skin flap, the entry point must be planned on the surface of the scalp instead of the skull bone. In summary, the application starts with taking a picture of a cranial CT or magnetic resonance imaging (MRI) from a DICOM viewing software or importing imaging material from a photo gallery. The image should be a coronal orientation with the lateral ventricles shown at the level of the anterior commissure. In moderate size ventricles, the coronal section should be in strict vertical orientation along the spine axis, while in narrow ventricles, the coronal plane should be individually tilted slightly anterior, optimally along the trajectory of the catheter placement. The image can be taken from a 3D MRI (e.g., MP-RAGE, 3D FFE, VIBE) or CT volume data set reconstructed by regular DICOM viewing software (e.g., Horos, Horos Project, New York, USA; Elements DICOM Viewer, Brainlab, Munich, Germany; RadiAnt DICOM Viewer, Medixant, Poznań, Poland; or MERLIN, Phönix-PACS, Freiburg, Germany) in order to visualize the coronal skin surface as close as possible to the entry site and foramen of Monro. The entry point in the sagittal plane from the nasion can now either be chosen as standard at 11.5 + / − 5 mm by default or measured exactly on the sagittal CT or MRI. The image is adjusted in size to an overlay and calibrated to the bitemporal diameter. This diameter should be determined with the DICOM viewing software in advance. Alternatively, 139 mm is an appropriate established approximation [12]. The next step is to define a paramedian entry point on the scalp surface by finger tapping on the screen with the help of an in-app implemented magnifying glass. Then, the two feet of the guiding tool’s base are virtually placed on the scalp surface bilateral to the entry point at the predefined radius of the guiding tool. The guide will then be presented as a schematic drawing. The preset orthogonal trajectory orientation of the tube is shown. A sliding bar allows the guide’s tube to virtually rotate around the entry point to shift the trajectory towards the ipsilateral ventricle. In addition, the finger is placed on the target within the ipsilateral ventricle to calculate the catheter length. Similarly, the finger is placed on the midline convexity of the skin, to calculate the entry point’s distance from midline.

### EVD placement

The patient is in supine position on an intensive care unit (ICU) or regular hospital bed in 30–50° of head elevation. For stabilization of the head against the bed, an emesis basin or donut head pad is placed beneath it and fixed with a conventional tape.

**Fig. 2:**
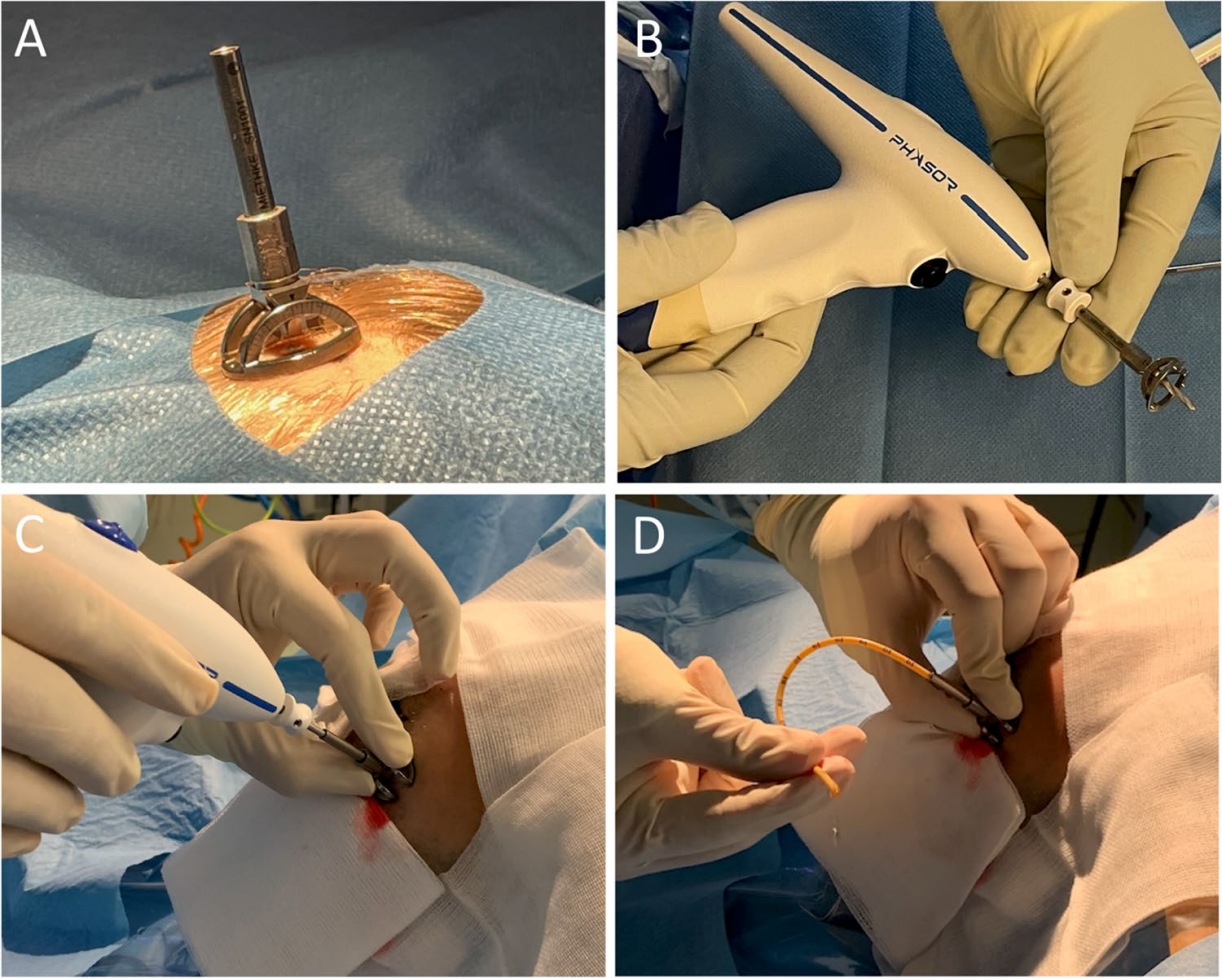
Technique used in human. **A** Placement of the catheter guide on the planned entry point on the scalp. **B** Electric power drill and catheter guide assembled. Note that the tip of the drill projects 15–20 mm beyond the bottom of the base ring of the catheter guide. **C** Insertion of the electric power drill through the tube of the catheter guide. **D** Insertion of the ventricular catheter through the pre-drilled burr hole with CSF flow

In concordance with the previously calculated parameters, the entry point is marked, and spray or wipe disinfected. A 5–10-mm straight skin incision is performed. The Thomale Guide is assembled with a 3.1-mm inner diameter tube, fastened with the locking nut to obtain the predetermined angulation and placed on the entry point. The disposable battery-powered 2.7-mm diameter drill (Phasor Health, Houston, USA) with an anti-plunge drill stop is introduced through the catheter guide until the tip reaches the skull bone, while the catheter guide is sturdily pressed with one hand onto the skin surface. A burr hole is made with the electric drill, and the catheter guide is removed out of the surgical field. The cranial dura mater is perforated with a standard 14-gauge venous cannula if needed. A standard silver impregnated EVD catheter with an outer diameter of 2.8 mm (VentriGuard®, Neuromedex, Hamburg, Germany) or a ventricular catheter with an outer diameter of 2.5 mm (Ventricular Catheter, Christoph Miethke, Potsdam, Germany) is inserted and guided through the pre-drilled burr hole to the calculated depth, indicated by the printed on scale, and then the guide wire is removed (Fig. 2). The ventricular catheter is tunneled for at least 4 cm from the insertion point subcutaneously, sutured to the skin and connected to an external CSF drainage system (VentrEX®, Neuromedex, Hamburg, Germany).

### Postoperative assessment

All patients received preoperative cranial CT or MRI scans and another postoperative scan on the following day after implantation. Quality of ventricular catheter position was evaluated using the GAVCA grading score [26]. The score implies number of puncture attempts, anatomical position, and rate of complete intraventricular position of the perforated part of the catheter tip. Retrospectively, radiological assessment, complications, length of EVD in place, volume of drainage, and possible follow-up treatment of CSF drainage were recorded.

Statistical analysis relied on Microsoft Excel, version 16.43, and figures were edited with Adobe Illustrator, version CC 2021.

Analysis on costs and revenues based on German market prices for medical products and the Diagnosis-related group (DRG) system as well as the Operation and Procedure Classification System (OPS), which serves as the German modification of the International Classification of Procedures in Medicine (ICPM).

## Results

### Baseline characteristics

A total of 12 patients (8 male, 4 female/11 adult, 1 pediatric) were operated. Patients had a mean age of 56 years (+ / − 18.9 years) with a range of 3 to 81 years. Main indication for EVD placement was aneurysmal subarachnoid hemorrhage (aSAH) followed by traumatic brain injury (TBI) and intracerebral hemorrhage (ICH). Mean preoperative fronto-occipital horn ratio (FOHR) was 0.4 (+ / − 0.06; range, 0.305–0.520), and fronto-occipital horn width ratio (FOHWR) was 0.22 (+ / − 0.07; range, 0.097–0.321) (Table 1).

**Table 1:** Clinical and radiological baseline characteristics

Patient	Age	Sex	Pathology	Preop FOHR	Preop FOHWR
1	3	m	HUS encephalopathy	0.400	0.160
2	66	m	Intracerebral hemorrhage	0.425	0.232
3	58	f	Traumatic brain injury	0.373	0.150
4	65	f	CSF shunt malfunction	0.520	0.314
5	62	f	Aneurysmal SAH	0.381	0.217
6	72	f	Hypoxic brain injury	0.455	0.273
7	46	m	Hypoxic brain injury	0.518	0.321
8	81	f	Meningoencephalitis	0.401	0.277
9	42	m	Intracerebral hemorrhage	0.362	0.097
10	64	f	Aneurysmal SAH	0.341	0.225
11	58	f	Traumatic brain injury	0.305	0.113
12	55	f	Aneurysmal SAH	0.394	0.245

*HUS* hemolytic-uremic syndrome, *CSF* cerebrospinal fluid, *SAH* subarachnoid hemorrhage, *FOHR* fronto-occipital horn ratio, *FOHWR* fronto-occipital horn width ratio

### Clinical and radiological outcome

Nine EVDs were implanted from the right and three from the left side. Three catheters were placed by using a lateral, eight catheters with a medial angulated trajectory, and one catheter with 0° angulation in relation to the perpendicular orientation towards the coronal calvarial slope at entry. The mean distance to midline was 26.6 mm (+ / − 2.3 mm; range, 22–31) with a mean implanted catheter length of 71 mm (+ / − 6.3 mm; range, 62–86) (Table 2).

**Table 2:** Navigation planning parameters of EVD trajectory

Catheter type	Side	Angulation (°)	Catheter length (mm)	Distance to midline (mm)
Miethke Ventricular Catheter	R	0	73	22
Neuromedex VentriGuard	R	13	72	28
Neuromedex VentriGuard	R	6	74	28
Neuromedex VentriGuard	L	12.5	76	31
Neuromedex VentriGuard	L	− 5	62	25
Neuromedex VentriGuard	L	− 6.5	72	27
Miethke Ventricular Catheter	R	8.5	86	26
Miethke Ventricular Catheter	R	4	67	29
Miethke Ventricular Catheter	R	3.5	70	25
Miethke Ventricular Catheter	R	3.5	64	26
Miethke Ventricular Catheter	R	− 1	66	26
Miethke Ventricular Catheter	R	3.5	70	26

Mean length of stay of an EVD was 6.3 days (+ / − 2.8 days; range, 3–13) with a mean drainage volume of 41.3 ml per day (+ / − 37.1 ml/d; range, 1.7–110.5), while in seven patients mainly ICP monitoring rather than CSF relief was indicated.

**Fig. 3:**
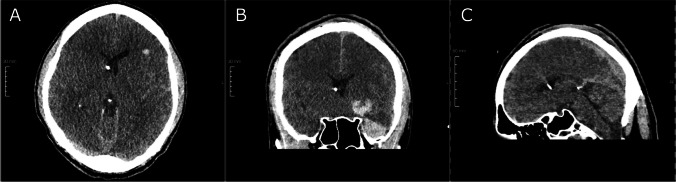
Representative postoperative cranial CT demonstrating an optimally placed EVD. Axial (**A**), coronal (**B**), and sagittal (**C**) cranial CT slice of a patient with narrow ventricles and intracerebral hemorrhage (ICH) at the level of the EVD tip

Five patients were implanted with a 2.8-mm EVD catheter (VentriGuard®, Neuromedex, Hamburg, Germany) and seven patients with a 2.5-mm catheter (Ventricular Catheter, Christoph Miethke, Potsdam, Germany) with a mean operating time of 25.75 min (+ / − 14.97 min; range, 10–40) including patient positioning, sterile table setup, preparation and draping in sterile fashion, and cleanup. All catheters were successfully placed at the first puncture attempt. None of the patients showed incorrect catheter placement evaluated with the GAVCA grading score. Eight patients showed optimal (grade I), and four patients showed intermediate catheter positions (grade II), while no grade III or IV catheter positions were observed. Anatomically, ten catheters were placed in the ipsilateral ventricle, and two had their tips in the third ventricle, while perforation holes showed entirely intraventricular location. The selection of the catheter’s outer diameter showed impact on the quality of catheter positions. After switching to the smaller catheter diameter, all catheters showed an optimal position in the postoperative scans (Fig. 3). During follow-up, two EVDs were converted into a ventriculoperitoneal shunt. Due to elevated CSF cell count and lactate values on postoperative day 5 without pathogen detection, antibiotic treatment was required before shunt insertion was done one week afterwards. Another patient had subdural hematoma with a thickness of 8 mm without necessity of intervention, and one EVD catheter was dislocated during transport from the ICU to the CT scanner on day five after implantation (Table 3).

**Table 3:** Clinical and radiological outcome

Length of stay (d)	Drainage volume (ml/d)	Infection	Hemorrhage	GAVCA grade	Anatomical position
6	1.7	No	No	2	3rd ventricle
4	6.3	No	No	1	Ipsilateral
4	3.8	No	No	2	Ipsilateral
3	85.7^†^	No	No	2	3rd ventricle
5	16.4	No	No	1	Ipsilateral
4	6.8	No	Yes	2	Ipsilateral
6*	79.3	No	No	1	Ipsilateral
5	82.0	No	No	1	Ipsilateral
8	24.9	No	No	1	Ipsilateral
13	110.5^†^	Yes	No	1	Ipsilateral
9	53.4	No	No	1	Ipsilateral
8	25.4	No	No	1	Ipsilateral

^*^Catheter replacement due to dislocation during CT transport

^†^Ventriculoperitoneal shunt conversion

### Cost and revenue analysis

Costs of medical products for standard bedside EVD insertion totaled up to 315.82 EUR compared to 347.60 EUR for navigated bedside EVD insertion. The ventricular catheter guide instrument was reusable, and the one-time acquisition cost was 1.2 TEUR, while the associated software application was free of charge. Revenues for EVD insertion and seven days of ICP monitoring summed up to 2.6 TEUR.

## Discussion

Several techniques have been developed to increase EVD implantation accuracy to avoid misplacement and associated complications like revisions, ICH, or neurological deficits, frequently associated with freehand technique in emergency situations. Here, we present a mobile health–assisted navigation technique using an adjustable catheter guide in combination with a battery-powered single-use power drill, which allows for precise bedside EVD implantation with reasonable time and cost expenditure.

The application of ventricular catheter guides as opposed to other techniques, such as fluoroscopy or computer tomography (CT), frameless stereotaxy, ultrasonography, neuroendoscopy, electromagnetic neuronavigation, and robotics, lies in its efficiency in terms of costs and time efforts. Previously, a simple guidance technique was suggested by Ghajar implementing a rectangular insertion angle towards the skull convexity in all planes at an entry point of 100 mm from the nasion and 30 mm lateral to the midline [10, 31, 32]. In comparison, the Thomale Guide enables ventricular catheter insertion with a rectangular sagittal angulation and an individual angulation in the coronal plane and has proven to be simple, consistent, and safe in improving ventricular catheter placement quality [21, 26].

In terms of technical considerations, we experienced some pearls and pitfalls, which are additionally worth mentioning. Since the planning of the entry point must be done from the scalp instead of the bony skull surface, as described previously for the operating room setting, adaptation especially in the coronal CT/MRI imaging is necessary to delineate the contrast of the skin surface for the virtual planning procedure. In the bedside setup, placing the ventricular catheter guide onto the relatively flexible scalp surface is a bit more susceptible to instability. Special care must be taken before starting to drill, as one hand has to remain solidly on the catheter guide until the burr hole is safely completed. Careful adjustment of an anti-plunge drill stop and strict maintenance of sterile conditions should be guaranteed to minimize intracranial hematoma and CSF infection as possible complications. In this study, only electric single-use power drills with a diameter of 2.7 mm were available, which lead to problems with the insertion of standard 2.8-mm EVD catheters (VentriGuard®, Neuromedex, Hamburg, Germany), as they tend to pass the burr hole only with increased physical effort. In the further course of the learning curve, a modification to a 2.5-mm diameter ventricular catheter (Ventricular Catheter, Christoph Miethke, Potsdam, Germany) was performed after the first five patients, which smoothened the procedure relevantly and lead to faster and better placement accuracy.

In CSF diverting shunts, optimal ventricular catheter position has been shown to be one of the most important factors to ensure functionality, otherwise leading to malfunction and operative revisions [5, 30]. The described ventricular catheter guiding technique was already successfully implemented in shunt implantation. However, since the mobility and flexibility of this technique presents its major advantage, the use for EVDs seems to be even more striking. The drawback until today for a bedside setup was a missing combination of the drilling procedure with the placement of the ventricular catheter guide. Application of an electric drill compared to a classic hand drill allows one-handed usage and thereby application of this technique by one surgeon without assistance. Moreover, less vibration is provoked by an electric drill compared to a hand drill in order to prevent distortion of the EVD trajectory.

Thus, we described for the first time the guided drilling procedure with the single-use electric drill fitting the tubing of the catheter guide and enabling the burr hole being oriented in the planned trajectory for accurate catheter placement. This enables to finally guide the catheter through the burr hole towards the ipsilateral ventricle and apply better reliability as a routinely applicable procedure. We were able to show feasibility of this technique to enhance EVD placement quality at reasonable time and cost efforts. Better placement reliability of EVDs is supposed to raise CSF drainage functionality as well as ICP measurement accuracy. Since this is the aim of the described technique, it will enhance the EVD placement indication in patients with intracranial hypertension, narrow ventricles, and/or midline shift like in TBI, where often intraparenchymal ICP monitoring is established as an alternative. Previously, this has been described to be superior in terms of clinical outcome [15], presenting fewer patients in need of decompressive craniectomy and improved neurological recovery, possibly influenced by an added therapeutic option of CSF drainage for ICP control and avoidance of secondary brain injury and herniation [28]. Additionally, application of medication instillation, e.g., for intraventricular lysis in intraventricular hemorrhage or application of antibiotic agents in CSF infections, could be positively impacted by optimized catheter position.

## Limitations

As this study was conducted as a clinical case series, there are some limitations. There is a small sample size from a single center, which reduces generalizability due to reduced power of data. Then, a selection bias might be introduced due to unsystematic patient inclusion within the department, depending on the neurosurgeon on call. Also, an equivalent comparison group would be desirable to investigate actual relevant differences to traditional EVD implantation, especially regarding clinical outcome.

## Conclusion

Bedside navigated EVD implantation, with the use of a mobile health–assisted, ventricular catheter guide in combination with a fitting electric single-use drill is an easy, safe, and reliable technique, resulting in accurate catheter position and functionality at low efforts.

## Data Availability

Supporting data and material are available upon request.

## Abbreviations

EVD: External ventricular drain
ICP: Intracranial pressure
CSF: Cerebrospinal fluid
CT: Computer tomography
MRI: Magnetic resonance imaging
ICU: Intensive care unit
aSAH: Aneurysmal subarachnoid hemorrhage
TBI: Traumatic brain injury
ICH: Intracerebral hemorrhage
FOHR: Fronto-occipital horn ratio
FOHWR: Fronto-occipital horn width ratio
